# Epstein-Barr Virus is Associated with Gastric Cancer Precursor: Atrophic Gastritis

**DOI:** 10.7150/ijms.71820

**Published:** 2022-05-21

**Authors:** Kai Zhao, Yu Zhang, Suhong Xia, Lina Feng, Wangdong Zhou, Mingyu Zhang, Ruonan Dong, Dean Tian, Mei Liu, Jiazhi Liao

**Affiliations:** Department of Gastroenterology, Tongji Hospital of Tongji Medical College, Huazhong university of Science and Technology, Wuhan 430030 Hubei Province, China

**Keywords:** *Helicobacter pylori*, Epstein-Barr virus, atrophic gastritis, gastric function

## Abstract

**Background:** About 10% of gastric cancer (GC) has been described to be Epstein-Barr virus (EBV) positive. Previous researches have described the association between EBV and GC. However, the association of EBV with atrophic gastritis (AG) is underrecognized. Our study aimed to investigate the relationship between EBV and AG and assess the influence of EBV on gastric function.

**Methods:** A total of 468 pathologically-confirmed chronic gastritis patients underwent circulating EBV DNA test, include 271 non-atrophic gastritis (NAG) and 197 AG patients.

**Results:** In this study, *H. pylori* infection rate was 33.3%, EBV infection rate was 40%, and co-infection rate was 15%. The EBV DNA-positive was significantly associated with AG (*P*=0.031, OR= 1.509, 95% CI 1.037-2.194), especially in *H. pylori*-negative subjects (*P*=0.044, OR=1.619, 95% CI 1.012-2.589). EBV DNA-positive patients had a lower pepsinogen I (PG I) / pepsinogen II (PG II) ratio (PGR) than EBV DNA-negative patients (*P*=0.0026), especially in the AG subgroup (*P*=0.0062). There was no significant association between EBV and *H. pylori* co-infection with increased risk of AG (*P*>0.05).

**Conclusion:** EBV infection significantly increased the risk of AG, especially in *H. pylori*-negative patients. The circulating EBV DNA had a potential in predicting the risk of atrophic gastritis.

## Introduction

Gastric cancer (GC) is one of the most common malignancies worldwide, and it is the fourth leading cause of cancer-related death 1. According to Lauren's classification, two histological subtypes of GC can be distinguished: intestinal and diffuse 2. About 90% of GC belong to intestinal-type GC, which represents the outcome of the inflammation-atrophic-metaplasia-dysplasia-carcinoma sequence, known as the Correa cascade 3. GC is a multifactorial disease, where many factors can influence its occurrence and development, include environmental, genetic, and infective factors 4. Regarding infective factors, *Helicobacter pylori* (*H. pylori*) and Epstein-Barr virus (EBV) have been well accepted as class I carcinogens and associated with GC occurrence and development 5.

EBV is a herpesvirus that favors human B lymphocytes 6. More than 90% of adults have been infected by EBV, and it is asymptomatic in the majority of carriers. EBV is closely associated with a variety of human malignant tumors, such as Burkitt's lymphoma, nasopharyngeal cancer, Hodgkin's disease, and GC, et al. 7 About 10% of GC has been described to be EBV-positive^8^. Many researches have described the association between EBV and GC 9. Atrophic gastritis (AG) is considered to be gastric precancerous diseases, which are independent risk factors of GC and provide background for the possible development of GC 10. Previous literature mentioned that the virus is localized in the atrophic epithelium in EBV-positive GC patients 11, and the lesions of EBV-related GC occur in the middle area near the atrophic boundary 12. EBV infection is also associated with an increased risk of GC and its precursor AG. Most patients who were EBV-positive had moderate AG 13, 14. These results suggested that EBV plays an important role in the development of GC. However, the association of EBV with AG is underrecognized. Thus, it would be greatly essential to identify the relationship between EBV and gastric precancerous diseases, and be beneficial for GC prevention, treatment, and block the progression of precancerous diseases.

The EBV detection methods include serological tests (anti-VCA IgM, anti-VCA IgG, anti-EBNA 1 IgG, and anti-EA(D) IgG) and circulating EBV DNA 15. EBV DNA and viral load can be detected by PCR, which has been already applied to examine the association between the EBV DNA and clinical courses of EBV-associated diseases using peripheral blood 16-18. EBV DNA is more sensitive and specific than serological methods 19. Based on this background, we applied real-time Q-PCR (RT-qPCR) to detect the copy number of EBV DNA using peripheral blood.

This study aimed to investigate the relationship between EBV with AG and to assess the influence of EBV on gastric function.

## Materials and Methods

### Study design and patients

This study enrolled 468 patients who underwent upper digestive endoscopy in the department of Gastroenterology of Tongji Hospital (Wuhan, China) from 2016 to 2021. The symptoms include epigastric pain, abdominal distension, heart burn, regurgitation, and nausea. Inclusion criteria for this study: 1) age from 18-80 years; 2) non-atrophic gastritis (NAG) and AG; 3) complete ^13^C urease breath test (^13^C-UBT), circulating EBV DNA and gastric function indicators such as pepsinogen I (PG I), pepsinogen II (PG II) and calculate the ratio of pepsinogen I and II (PGR). Exclusion criteria were as follows: 1) the history of gastric dysplasia or cancer, nasopharyngeal carcinoma or other malignant tumors; 2) autoimmune atrophic gastritis; 3) other types of chronic gastritis: such as NSAIDS-related gastritis, Eosinophilic gastritis, Menetrier disease, et al. The study was conducted in accordance with the Declaration of Helsinki (as revised in 2013). The study was approved by the Ethical Committees of Tongji Hospital (TJ-IRB20220534).

### Endoscopy and histopathological diagnosis

All subjects underwent gastroscopy with two biopsies taken from the gastric antrum and two biopsies taken from the gastric corpus. The gastroscopy of all patients was performed by experienced endoscopists using an Olympus endoscopy (XQ260, XQ290, Tokyo, Japan). Histopathological diagnosis was performed by experienced digestive pathologists, who observed the changes of atrophic. Gastric inflammation and atrophy were diagnosed according to the Chinese Consensus on Chronic Gastritis (2017, Shanghai, China). Subjects with multiple lesions were diagnosed based on the most severe lesion.

### Assessment *of H. pylori infe*ction

*H. pylori* infection was detected by ^13^C-UBT, which is widely considered as one of the most accurate tests for *H. pylori* infection detection. Proton pump inhibitors, acid-suppressive drugs, and antibiotics were not permitted for 2 weeks prior to the ^13^C-UBT. The ^13^C-UBT was detected using a urea ^13^C capsule breath test kit (KEADWAY, Shenzhen, China). After fasting 2 hours, the subjects were given a tablet with ^13^C and collected the breath samples were at 0 min and 30 min. A ^13^C-Breath Test Analyzer (HeliFANplus, Germany) aided in data analysis. Positivity was defined as a value >4.0, indicating the presence of *H. pylori* infection.

### Quantitative analysis of circulating EBV DNA load

Peripheral venous blood sample (5ml) was collected into EDTA-containing tubes from all subjects. The RT-qPCR system was developed to detect EBV DNA. This was used to identify the *Bam* HI-W region of the EBV genome. The Bam HI-W consisted of the amplification primers W-44F (5′-CCCAACACTCCACCA CACC-3′) and W-119R (5′-TCTTAGGAGCTGTCCGAGGG-3′) and the dual-labeled fluorescent probe W-67T (5′-[FAM] CACACACTACACACACCCACCCGTCTC [TAMRA]-3′).

Fluorogenic PCR reactions were set up in a reaction volume of 50ul using the TaqMan PCR core Reagent Kit (TGFQ01-001, Targene, Guangzhou, China). Thermal cycling was initiated with a 2-minute denaturation step at 93 °C, followed by 10 cycles at 93 °C for 45 seconds and 55 °C for 1 minute, and 30 cycles at 93 °C for 30 seconds and at 55 °C for 45 seconds. Duplicate samples were analyzed, and the mean quantity of each duplicate was used for further concentration calculations. Multiple negative blanks were included in every analysis. In our study center, a cutoff value of 500 copies/ml was set according to the lower limit of detection. EBV DNA ≥ 500 copies /ml was defined as EBV-positive.

### Detection of serum PG I and PG II

Peripheral venous blood sample (5ml) was collected from each eligible subject. Serum PG I and PG II were detected in the Tongji Hospital (Wuhan, China). Serum levels of PG I and PG II were carried out by ELISA (BOTHIT Plc, Helsinki, Finland).

### Statistical analysis

SPSS 26.0 was used for the statistical analysis (SPSS, Chicago, IL, USA). Descriptive statistics were based on the total number (%) and expressed as mean ± standard deviation. Chi-squared or Fisher tests were used for quantitative data analysis. To evaluate the risk provided by EBV and *H. pylori* to AG, the odd rates (ORs) were estimated. The group of EBV and *H. pylori* double-positive patients was compared with the group infected with only *H. pylori* or EBV, A two-sided *P*-value of <0.05 was considered statically significant.

## Results

### Baseline Characteristics of the Subjects

A total of 468 patients with a median age of 48.1 ± 8.7 years were enrolled in our study. The baseline features of all patients are shown in Table 1. The study subjects were classified as 271 (57.9%) NAG patients and 197 (42.1%) AG patients. *H. pylori* infection rate was 33.3%, EBV infection rate was 40%, and co-infection rate was 15%.

### Distribution Characteristics of Circulating EBV DNA of the Subjects

We analyzed the distribution of circulating EBV DNA based on age, smoking, drinking, gender, *H. pylori* infection status, and gastritis classification (Table 2). We found that the EBV DNA-positive were correlated with the older group (≥50 years) and AG group (*P*<0.05). No significant difference was found between EBV and gender, smoking, drinking and *H. pylori* infection status (*P*>0.05).

### Association between Circulating EBV DNA and AG Risk

We found that the positive rate of EBV DNA was significantly higher in the AG group than NAG group (45.7% vs 35.8%), and EBV DNA-positive could increase the risk of AG by 1.509-fold (*P*=0.031, 95% CI 1.037-2.194) (Table 2). Stratified analysis was further performed based on age, gender, and *H. pylori* infection status. We found that the contribution of EBV DNA to AG risk was statistically significant in the *H. pylori* subgroups, but not in the age and gender subgroups (*P*>0.05). In the *H. pylori*-negative subgroups, the risk of AG was increased by 1.619-fold (44.5% vs 33.2%, *P*=0.044, 95% CI 1.012-2.589) (Table 3).

### Association between Circulating EBV DNA and Pepsinogens

**Figure 1:**
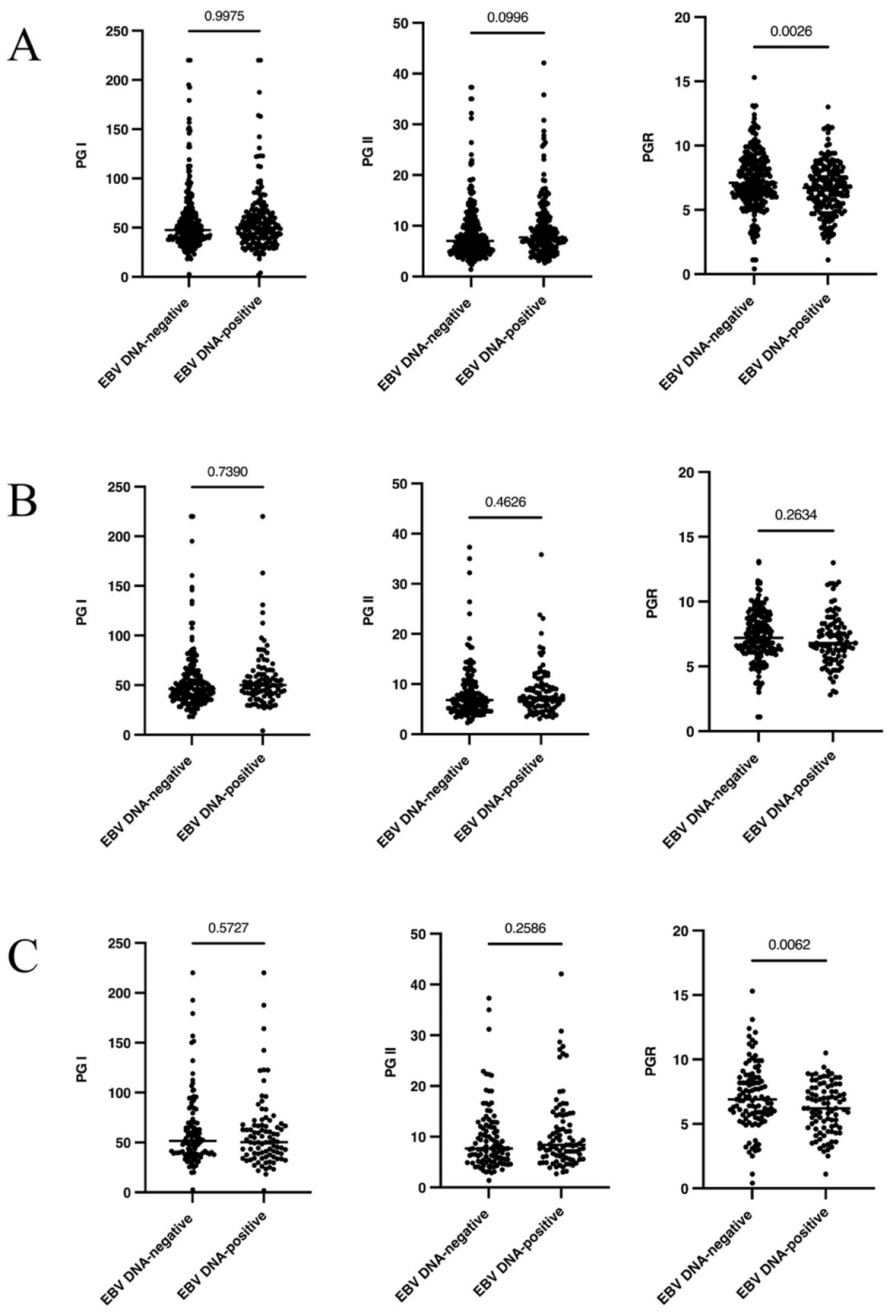
**The association between circulation EBV DNA and gastric function indications (PG I, PG II and PGR) in different EBV carriers**. (A): The overall population. (B): In NAG subgroup. (C): In AG subgroup. *H. pylori*:* Helicobacter pylori*; EBV: Epstein-Barr virus; NAG: No-atrophic gastritis; AG: atrophic gastritis.

To analyze whether circulating EBV DNA status was associated with gastric function, gastric function indicators were measured in all patients. Compared with EBV DNA-negative patients, the level of serum PGR was significantly decreased in EBV DNA-positive patients (*P*=0.0026), while no significant difference was observed in PG I and PG II (*P*>0.05) (Figure 1A). Stratified analysis was further performed based on gastric mucosal status. In the NAG subgroup, the serum gastric function indicators were not significantly variated between EBV DNA-positive with EBV DNA-negative patients (*P*>0.05) (Figure 1B). In the AG subgroup, the serum PGR also significantly decreased in EBV DNA-positive patients compared with EBV DNA-negative patients (*P*=0.0062) (Figure 1C).

### Association between* H. pylori*/EBV Co-infection and AG Risk

To explore the interaction among AG related infective factors, the relationship between circulating EBV DNA and *H. pylori* was analyzed. There was no difference between EBV DNA and *H. pylori*, regardless of whether the overall population or the subgroups (*P*>0.05) (Table 4). However, we found that EBV DNA-positive increased the risk of AG by 1.619-fold in the *H. pylori*-negative subgroup (Table 3) and the serum PGR also significantly decreased in EBV DNA-positive patients of the AG subgroup (Figure 1C).

So as to analyze whether there is more risk of getting gastric precancerous disease by infection of the two both pathogens, we compared the group of EBV and *H. pylori* co-infection (EBV + /*H. pylori* +) patients with the group infected with only *H. pylori* or EBV (EBV + /*H. pylori* - or EBV - /*H. pylori* +). Whether EBV + /*H. pylori* + vs EBV + /*H. pylori -* or EBV + /*H. pylori* + vs EBV - /*H. pylori* +, there was no association with AG (*P*>0.05) (Table 5).

## Discussion

The prevalence of EBV varied in patients with NAG and AG in the studies, which is directly related to race, geography, and detection methods. Cárdenas-Mondragón et al. 20 found that in patients with chronic gastritis, the infection rate of EBV was 92%, *H. pylori* was 85.8%, and the co-infection rate was 77.7%; in the gastric precancerous disease, the EBV infection, *H. pylori* infection and co-infection rate were 97.9%, 89.3%, and 88.2%. A study of EBV detection in Mexico 21 showed that the rate of EBV and *H. pylori* infection was 69.8% and 48.1%, and the co-infection was 25.4%. In the northern Chinese population 10, the infection rate of EBV was 35.2%, (30.5% with chronic gastritis, 40% with AG, and 38.6% with GC). The incidence of EBV associated gastric cancer is 30.8% in Guangzhou 22. In our study, we found that the infection rate of EBV in patients was 40.0%, include 35.8% in patients with chronic NAG and 45.7% in patients with AG, which is similar to Chinese studies.

Long lasting inflammation triggers serious damage to the gastric epithelium, increasing the risk to develop precancerous lesions, which in turn increase the risk to end up with a life-threatening GC. More than 90% of GC is related to persistent inflammation. EBV and *H. pylori* are both considered the main risk factors for chronic inflammatory responses triggering tissue damage 23. Previous literature mentioned that in EBV-positive gastric carcinoma, the virus was localized in the atrophic epithelium 12. Nevertheless, Hungermann et al. 24 found evidence that EBV infected epithelial cells of AG mucosa with a relatively low frequency, which is not an early event in GC. Cárdenas-Mondragón et al. 20 study showed that EBV-positive conferred 3.5-fold increased AG risk. Wang et al. 10 found that EBV infection could increase AG risk, especially in the younger and female subgroups. In our study, we found that EBV positive increased the risk of AG by 1.509-fold (*P*=0.031, 95% CI 1.037-2.194).

There were contradictory results in the effect of EBV and *H. pylori* co-infection in gastroenterology diseases. Su et al. 25 found that EBV was negatively correlated with *H. pylori* infection, and there may be antagonism effects between the infection of EBV and *H. pylori* in GC. Other studies suggested that EBV was positively correlated with *H. pylori*, EBV and *H. pylori* co-infection to induce severe inflammation 26, 27, increasing the risk of progression to intestinal-type GC and gastric precancerous diseases 20, 28. Some studies found that EBV was not associated with *H. pylori* infection in GC 10, 21, 29. In our study, there was no significant correlation between EBV and *H. pylori* (*P*>0.05*)*. EBV and *H. pylori* co-infection did not promote the development of gastric mucosa to gastric precancerous disease. Only in *H. pylori*-negative patients, EBV DNA-positive patients increased AG risk by 1.619-fold. Nerva Dursun et al. 30 also found that atrophy was frequently observed in EBV-positive and *H. pylori*-negative cases of gastritis.

*H. pylori* infection was considered to be the prominent cause of GC, which spreads from contaminated food. *H. pylori* was mainly spread transmitted orally and adhered to the gastric epithelial cells, leading to oxidative stress, toxin, and necrosis of cells, which further lead to chronic inflammation epigenetic modification, and mutation 28. However, EBV spreads mainly by the oral route through contact with saliva. More than 90% of adults have EBV in the latent stage in B cells 31. The infected B cells sometimes enter the lytic cycle, producing virus particles that can spread to other hosts. Stable EBV infection and latent EBV gene expression are favorable for promoting the transformation of pre-invasive nasopharyngeal epithelial cells into carcinoma 32. Moreover, similar to the mechanism of *H. pylori* induced GC, EBV may indirectly induce chronic inflammation during its viral reactivation cycle by recruiting high levels of immune cell infiltration and thereby promoting tissue damage 33. Several researched have shown that the cooperation of infectious agents may exacerbate their effect. EBV DNA load in *H. pylori*-positive individuals was significantly increased, which suggested that *H. pylori* might play a role in regulating the transformation of EBV to the cleavage stage 34. *H. pylori* Cag A was demonstrated associated with the development of GC. Saju et al. 35 discovered that host protein SHP 1 interacts with *H. pylori* Cag A protein and dephosphorylates Cag A, which antagonized the Cag A oncogenic activity. However, EBV co-infection results in methylation of the host SHP 1 and keeps Cag A phosphorylation and thus may increase the oncogenic potential of Cag A. Fekadu et al. 36 found that *H. pylori* was exposed to the principal EBV receptor, CD21, in negative gastric epithelial cells, which could induce the expression of EBV receptors EphA2 and NMHC-IIA, and promote the EBV infection. And EphA2 or NMHC-IIA siRNA knockdown, EBV infection was significantly decreased. These results suggest that EBV and *H. pylori* may have some synergistic effects on the development of GC. In the future, there should be some studies and experiments with a more rigorous design, to confirm the mechanism of how EBV and/or *H. pylori* infection interacts.

Pepsinogen (PG) and gastrin (G17) are effective indicators that reflect gastric function and screen of GC and its precursor 37. In our study, we explored the relationship between EBV infection and gastric function. We found that the serum PGR in EBV DNA-positive patients was decreased (*P*<0.05), while other indicators showed no significant difference (*P*>0.05). We further found that in the AG subgroup, the serum PGR in the EBV DNA-positive group was lower than that in the EBV DNA-negative group (*P*<0.05). Su et al. 25 found that EBV-positive patients had significantly higher levels of PGI and PGR than EBV-negative patients (*P*<0.05). Wang et al.^10^ found that the level of PGR in the EBV-positive patients was significantly lower than that of EBV-negative patients (*P*<0.05), while other indicators showed no significant difference (*P*>0.05). And the serum PGR in the EBV DNA-positive group was lower in the AG subgroup (*P*<0.05). The differences in the results may be caused by the different diagnostic criteria and detection methods of EBV in different institutes. Reduction in the PGR demonstrated a strong association with the development of AG and GC. In our study, we found preliminary evidence that EBV DNA was correlated with a low PGR, especially in AG subgroup. These results may provide research clues for confirming that the EBV infection may promote the occurrence of AG.

There were also some research limitations in this study. Firstly, geographical differences in EBV positive had been observed. Our study was a single-center study, we should enroll more patients from different hospitals and regions for research in the future. Second, we enrolled every patient with NAG or AG who came to the institute for diagnosis in a convenient sample instead of paired selection. Lastly, in situ hybridization (ISH) of tissue, a gold benchmark in the determination of EBV infection, was lacking in this study. We should comprehensively evaluate EBV infection, include circulating EBV DNA, multiple specific antibodies for EBV antigens, and ISH, et al.

## Conclusion

Given the above, this study showed that the positive EBV DNA was associated with an increased risk of gastric precancerous disease, which was more notable in *H. pylori*-negative individuals than *H. pylori*-positive individuals. EBV DNA-positive patients demonstrated a lower serum PGR, which was more distinct in AG patients.

## Figures and Tables

**Table 1:** The baseline characteristics of patients with NAG and AG

Value	Total (n=468)
Age	48.1 ± 8.7
<50 years	248 (53.0%)
≥50 years	220 (47.0%)
BMI	24.8 ± 3.8
Smoking	91 (19.5%)
Drinking	59 (12.6%)
Gender	
Male	333 (71.2%)
Female	135 (28.8%)
Gastric lesion	
NAG	271 (57.9%)
AG	197 (42.1%)
*H. pylori* positive	156 (33.3%)
EBV positive	187 (40%)
*H. pylori* and EBV co-infection	70 (15.0%)

*H. pylori*:* Helicobacter pylori*; EBV: Epstein-Barr virus; NAG: No-atrophic gastritis; AG: atrophic gastritis.

**Table 2:** The characteristics on quantitative of circulating EBV DNA

Variable	EBV-Negative	EBV-Positive	*P*-value	OR (95% CI)
Age				
<50 years	162 (65.3%)	86 (34.7%)	0.013	1.599 (1.102-2.320)
≥50 years	119 (54.1%)	101 (45.9%)		
Smoking	54 (19.3%)	37 (19.8%)	0.894	1.032 (0.648-1.646)
Drinking	34 (12.1%)	25 (13.4%)	0.696	1.117 (0.642-1.941)
Gender				
Male	196 (58.9%)	137 (41.1%)	0.412	0.842 (0.557-1.270)
Female	85 (63%)	50 (37%)		
*H. pylori* infection status				
Negative	195 (62.5%)	117 (37.5%)	0.125	1.357 (0.919-2.004)
Positive	86 (55.1%)	70 (44.9%)		
Gastritis classification				
NAG	174 (64.2%)	97 (35.8%)	0.031	1.509 (1.037-2.194)
AG	107 (54.3%)	90 (45.7%)		

*H. pylori*:* Helicobacter pylori*; EBV: Epstein-Barr virus; NAG: No-atrophic gastritis; AG: atrophic gastritis.

**Table 3:** The stratified analysis on association between circulating EBV DNA with AG risk

Variable		EBV-Negative	EBV-Positive	P-value	OR (95% CI)
Gender					
Male	NAG	123 (62.8%)	73 (37.2%)	0.084	1.477 (0.948-2.301)
	AG	73 (53.3%)	64 (46.7%)		
Female	NAG	51 (68.0%)	24 (32.0%)	0.175	1.625 (0.803-3.287)
	AG	34 (56.7%)	26 (43.3%)		
Age					
<50 years	NAG	109 (68.1%)	51 (31.9%)	0.211	1.411 (0.822-2.425)
	AG	53 (60.2%)	35 (39.8%)		
≥50 years	NAG	65 (58.6%)	46 (41.4%)	0.180	1.439 (0.845-2.451)
	AG	54 (49.5%)	55 (50.5%)		
*H. pylori* infection status					
Negative	NAG	129 (66.8%)	64 (33.2%)	0.044	1.619 (1.012-2.589)
	AG	66 (55.5%)	53 (44.5%)		
Positive	NAG	45 (57.7%)	33 (42.3%)	0.520	1.231 (0.654-2.315)
	AG	41 (52.6%)	37 (47.4%)		

*H. pylori*:* Helicobacter pylori*; EBV: Epstein-Barr virus; NAG: No-atrophic gastritis; AG: atrophic gastritis.

**Table 4:** The association between circulating EBV-DNA with *H. pylori* infection status

Variable	EBV-Negative	EBV-Positive	P-value
*H. pylori* infection status			
Negative	195 (62.5%)	117 (37.5%)	0.125
Positive	86 (55.1%)	70 (44.9%)	
NAG group			
Negative	129 (66.8%)	64 (33.2%)	0.155
Positive	45 (57.7%)	33 (42.3%)	
AG group			
Negative	66 (55.5%)	53 (44.5%)	0.690
Positive	41 (52.6%)	37 (47.4%)	

*H. pylori*:* Helicobacter pylori*; EBV: Epstein-Barr virus; NAG: No-atrophic gastritis; AG: atrophic gastritis.

**Table 5:** Association between *H. pylori*/EBV co-infection with AG risk

	NAG	AG	*P*-value	OR (95% CI)
*H. pylori* infection status				
Negative	193 (71.2%)	119 (60.4%)	0.014	1.622 (1.100-2.391)
Positive	78 (28.8%)	78 (39.6%)		
EBV DNA qualitative				
Negative	174 (64.2%)	107 (54.3%)	0.031	1.509 (1.037-2.194)
Positive	97 (35.8%)	90 (45.7%)		
*H. pylori* + /EBV + VS *H. pylori* + /EBV -	33 (42.3%)	37 (47.4%)	0.520	1.231 (0.654-2.315)
	45 (57.7%)	41 (52.6%)		
*H. pylori* + /EBV+ VS *H. pylori* -/EBV +	33 (34.0%)	37 (41.1%)	0.317	1.354 (0.748-2.452)
	64 (66.0%)	53 (58.9%)		

*H. pylori*:* Helicobacter pylori*; EBV: Epstein-Barr virus; NAG: No-atrophic gastritis; AG: atrophic gastritis.
